# Assessment of Disability among the Elderly in Xiamen of China: A Representative Sample Survey of 14,292 Older Adults

**DOI:** 10.1371/journal.pone.0131014

**Published:** 2015-06-30

**Authors:** Wei Chen, Ya Fang, Fanzhen Mao, Shichao Hao, Junze Chen, Manqiong Yuan, Yaofeng Han, Y. Alicia Hong

**Affiliations:** 1 State Key Laboratory of Molecular Vaccinology and Molecular Diagnostics, School of Public Health, Xiamen University, Xiamen, Fujian, China; 2 Key Laboratory of Health Technology Assessment of Fujian Province University, School of Public Health, Xiamen University, Xiamen, Fujian, China; 3 Health Promotion and Community Health Sciences, School of Public Health, Texas A&M Health Science Center, College Station, Texas, United States of America; University of Perugia, ITALY

## Abstract

**Background:**

The unprecedented number of elderly individuals in China presents a serious public health challenge. Limited data are available on the prevalence of disability or factors resulting in disability among the elderly in China.

**Objective:**

We aimed to assess the prevalence of disability and related risk factors among the elderly of Xiamen, China.

**Methods:**

A cross-sectional study was performed on individuals who were ≥60 years of age. The subjects were recruited by multi-stage sampling; a total of 14,292 valid questionnaires were received. Study measurements included activities of daily living (ADL), demographics, and health status. The ADL was assessed by the Katz Index Scale to evaluate disability. Chi-square tests and binary logistic regression were used to identify factors associated with disabilities.

**Results:**

Among the valid participants, 4.27% had at least one disability. Bathing was the most frequently reported disability and feeding was the least frequently reported disability. Disabilities were significantly associated with female gender, older age, unmarried status, living with family, urban residence, illiteracy, poor economic status, self-rated bad health, chronic illnesses, lower life satisfaction, bad mood, and feelings of loneliness.

**Conclusion:**

Functional disability among the elderly requires more public attention. Culturally appropriate policies and programs are also needed to address the care for the disabled elderly.

## Introduction

Population aging is a global concern that no longer only occurs in developed countries. China became an “aging society” around 2000, and has experienced an unprecedented process of aging in its population [1–3]. The “One Child Policy” adopted in 1979 has limited the crude birth rate. Additionally, life expectancies have increased and mortality rates have decreased; thus the percentage of elderly population has grown substantially in the past 30 years [4–6]. In 2013, there were nearly 202 million people who were ≥60 years of age and 132 million who were ≥65 years of age in China, accounting for 14.9% and 9.7% of the total population, respectively [7]. Population projections predict the proportion of individuals aged ≥60 years and aged ≥ 65 years in China will exceed 30% and 20% respectively by 2050 [3,4,8,9].

China has encountered the situation of “becoming old before becoming rich” [10], as a developing country with a large aging population. It faces a hefty challenge of caring for its elderly population, especially those with functional disabilities. By 2010, China had more than 10.8 million disabled elderly people, accounting for 6.25% of the total elderly population [11]. Once the elderly become disabled, their physical health declines along with their mental health; their quality of life deteriorates rapidly [12] and they require long-term care. Thus, millions of families face the stress and burden of taking care of these disabled elderly individuals, and billions in healthcare costs are needed to cover their long-term care [13].

Due to the rapidly increasing number of elderly and the large number of disabled elderly in China, it is urgent to study both the prevalence of elderly individuals with disabilities and key factors causing those disabilities. The latest national research in 2010 showed that the prevalence of elderly with disabilities was 6.25% [11]. Several studies explored factors impacting disabilities, such as residence [14,15] and household structure [16,17]. However, the previously mentioned studies were usually small-scale at the municipal level and national research did not investigate factors associated with disabilities. Under the current healthcare system in China, long-term care for elderly is administered at the municipal level. Therefore, we conducted the current study among the elderly in the Xiamen municipal area to assess the prevalence of elderly individuals with disabilities. We obtained this information from a large representative sample in order to explore related risk factors.

## Materials and Methods

### Study Site

The study was conducted in the Xiamen municipal area. Xiamen is located on the southeast coast of China, facing the East China Sea and Taiwan. As one of the first four “Special Economic Zones” in China, Xiamen began its economic reforms and opened up to the Western world in 1978. Xiamen is more economically developed than most other regions of China; its per capital GDP is $13,170 US dollars, ranking among the highest nationwide [18]. It is also known for its mild weather and has been voted one of the most livable cities in China. Xiamen is comprised of 6 districts, 38 sub-districts, and 479 communities. As of August 2013, a population of 1.94 million households was registered in Xiamen of which 264,624 were aged ≥60 years, accounting for 13.65% of the total registered population.

### Sampling process

A cross-sectional study was performed on individuals aged ≥60 years who registered their households for interviews. The initial sample size was calculated by the following formula (estimation for population probability) [19]:

$$n={\left\{\frac{Z_{\alpha /2}}{\text{arcsin}\left[\delta /\sqrt{P\left(1-P\right)}\right]}\right\}}^{2}.$$

*P* was set at 6.25%, according to the latest national survey mentioned previously [11]. *α* and *δ* were set at 0.05 and 0.10*P* (0.625%), respectively. Hence, the calculated sample size was 5,762 individuals. However, in order to establish a baseline database, we intended to recruit 5% of the overall registered elderly population in Xiamen; this number was much greater than 5,762. In August 2012, according to the data from the Xiamen Municipal Committee on Ageing, there were 261,043 registered people ≥60 years of age. Using the baseline recruitment value, 13,053 participants were recruited into the study. When considering the effectiveness of the returned questionnaires, we added 1,305 participants (10% of the baseline recruitment value) and expanded the sample size to 14,358 participants.

**Table 1**showed the determination procedure as related to sample size. Multistage probability sampling was performed in order to select participants. In the first stage, participants were enrolled and organized into six districts that were organized alphabetically from A to F. In the second stage, individuals were selected and placed into 38 sub-districts. In the third stage, individuals were placed into communities. One third of the communities were randomly selected from each sub-district; for a total of 173 included communities. Randomization of these communities was performed by computer-generated numbers. In the fourth stage, the individuals were conveniently selected from the community by the interviewers; control group characteristics were analyzed according to gender and age. For instance, suppose that among the qualified candidates in a single district, 20% were male and between 60~69 years of age with 200 participants expected for each community in this district. From this statistical analysis formula, each community investigated 40 (200×0.2) male individuals between 60~69 years of age.

**Table 1:** The distribution of elderly participants by multistage stratified sampling.

District (1)	Qualified candidates (2)	Total communities (3)	Sampled Communities^a^ (4) = (3)*1/3	Expected participants per community (5)	Expected participants (6) = (4)*(5)	Actual participants (7)	Valid participants (8)
A	100,502	96	35	150	5250	5248	5238
B	26,857	50	18	81	1458	1519	1518
C	27,375	59	21	72	1512	1514	1514
D	15,732	36	14	63	882	875	872
E	47,580	126	46	58	2668	2697	2695
F	42,997	112	39	63	2457	2520	2455
Total	261,043	479	173	-	14,227	14,373	14,292

^a^ If the number of communities in each sub-district were not divisible by three, the quotients were ceilinged. For example, there were 3 sub-districts (a, b and c) in D district, and the number of communities in a, b and c sub-district were 19, 4 and 13, respectively. The number of sampled communities in D district would be calculated as follow: 19*1/3+4*1/3+13*1/3≈7+2+5 = 14.

A total of 14,373 respondents were interviewed; the survey included two stages and lasted three entire months in 2013 (August 1^st^ to November 2^nd^, 2013). The first stage of the survey investigated the population. All participants were investigated from August 1^st^ to September 30^th^, 2013, and 11,256 questionnaires were completed during this time; these included 3,043 questionnaires containing the missing data and 74 invalid questionnaires (63 questionnaires were unreliable and 11 participants were ≤60 years of age). The second of the survey stage checked the data. An analysis of the returned surveys was conducted to check missing data from October 8^th^ to November 2^nd^, 2013; these surveys included 3,036 valid questionnaires (i.e., completed assessment of disability and >90% of other data) and 7 invalid questionnaires (>50% missing data). In the end, a total of 14,292 (99.44%) valid questionnaires were obtained.

### Measures

#### Assessment of disability

The extent of disabilities is usually measured by activities of daily living (ADL) [20,21]. A modified Katz Index Scale [14,22–24] was applied to measure the ADL in our study, which included the following six items: dressing, feeding, transferring (getting in/out of bed), walking (walking around inside), bathing, and toileting. Each item had three response choices: “completely independent”, “needing some help”, and “completely dependent”. If any answer was “completely dependent”, participants were defined as disabled; otherwise the participants were categorized as nondisabled. This scale had good reliability and was used by Lin et al [25] to assess functional status of elderly in China.

#### Other study variables

Demographic variables included the following: gender (male, female), age, marital status (married, unmarried), living arrangements (living alone, living with family), residence (urban, rural), education level (illiterate, primary, junior high school, senior high school, college and above), and personal economic status (income exceeded expenditure, balance, expenditure exceeded income). The following health status data were self-reported: health (good, fair, bad), chronic diseases, life satisfaction (good, fair, bad), mood (good, fair, bad) and feelings of loneliness (seldom/never, sometimes, often). The self-rated health variables (life satisfaction, mood, feelings of loneliness) were assessed by questions about the participants’ feelings during the last month of the survey.

### Data collection procedure

Four hundred investigators were trained with regards to standard operating procedures (SOP). A face-to-face interview was conducted at the participants’ homes. The questions could be answered by proxies if participants were unable to answer. It took between 15 to 20 minutes to complete each interview. No gifts or rewards were given to any participant. A return survey was conducted in order to check missing data if questionnaires were incomplete. All respondents participated voluntarily and gave their formal consent. The study protocol was approved by the School of Public Health, Xiamen University.

### Data analysis

First, some basic descriptive statistics were calculated, including mean and standard deviations for quantitative variables, and proportions for qualitative variables. Second, reliability of the modified Katz Index Scale was assessed by Cronbach’s alpha coefficient. Third, chi-square tests were performed to examine differences in disability prevalence among the variables mentioned above. Finally, forward binary logistic regression was applied to identify impact factors. Entered variables had a *P*-value of ≤0.05 and removed variables had a *P*-value of ≥0.10. Data entry was performed independently and discrepancies were checked by two trained research assistants using Epidata software version 3.1 (Epidata Association, Odense, Denmark). All statistical analyses were conducted using IBM SPSS software version 21.0 (IBM SPSS Statistics for Windows, Version 21.0. Armonk, NY, United States). A value of *P*< 0.05 was considered statistically significant.

## Results

### Demographic characteristics and health status of participants

The demographic characteristics and health status of all valid participants are shown in **Table 2**. There were slightly less men than women (48.19% vs.51.81%). Their ages ranged from 60 to 103 years with a mean age of 71.49±8.34 years. The proportion of participants decreased as age increased; there were 48.22% of participants between 60~69 years of age. The proportion of married participants and single participants were 69.56% and 11.07%, respectively. Slightly more elderly participants were living in rural (51.08%) than in urban (48.92%) residences. There were 63.78% of the participants who had only attained primary or lower-level education. The proportion of the elderly in poor economic status and self-rated poor health were 27.47% and 17.97%, respectively. Among study participants, 63.40% suffered from chronic diseases, 54.31% were satisfied with their lives, 47.58% had good mood, and 61.8% seldom felt feelings of loneliness.

**Table 2:** Characteristics of the elderly and disability prevalence.

	*n* (%)	Disabled	Prevalence (%)	*χ* ^2^	*P*-value
**Gender (*N* = 14,292)**					
Male	6888 (48.19)	228	3.31	29.87	0.000
Female	7404 (51.81)	382	5.16		
**Age (years) (*N* = 14,292)**					
60~69	6882 (48.22)	99	1.44	537.95	0.000
70~79	4646 (32.51)	181	3.90		
80~above	2754 (19.27)	330	11.98		
**Marital status (*N* = 14,268)**					
Married	9925 (69.56)	273	2.75	185.21	0.000
Unmarried	4343 (30.44)	337	7.76		
**Living arrangement (*N* = 14,285)**					
Alone	1582 (11.07)	61	3.86	0.72	0.395
With family	12,703 (88.93)	548	4.31		
**Residence (*N* = 14,292)**					
Urban	6992 (48.92)	301	4.30	0.05	0.831
Rural	7300 (51.08)	309	4.23		
**Education (*N* = 14,204)**					
Illiterate	4659 (32.80)	295	6.33	90.70	0.000
Primary	4401 (30.98)	179	4.07		
Junior high school	2689 (18.93)	63	2.34		
Senior high school	1579 (11.12)	42	2.66		
College and above	876 (6.17)	22	2.51		
**Economic status (*N* = 14,284)**					
Income exceeded expenditures	2938 (20.57)	45	1.53	506.71	0.000
Balance	7422 (51.96)	155	2.09		
Expenditures exceeded income	3924 (27.47)	410	10.45		
**Self-rated health (*N* = 14,291)**					
Good	4820 (33.73)	7	0.14	2174.66	0.000
Fair	6902 (48.30)	61	0.88		
Bad	2569 (17.97)	542	21.10		
**The number of chronic diseases (*N* = 14,286)**					
0	5228 (36.60)	73	1.40	352.74	0.000
1	4304 (30.13)	162	3.76		
2	2752 (19.26)	151	5.49		
≥3	2002 (14.01)	224	11.19		
**Life Satisfaction(*N* = 14,287)**					
Good	7759 (54.31)	146	1.88	612.41	0.000
Fair	5701 (39.90)	299	5.24		
Bad	827 (5.79)	164	19.83		
**Mood(*N* = 14,284)**					
Good	6797 (47.58)	143	2.10	741.87	0.000
Fair	6970 (48.80)	324	4.65		
Bad	517 (3.62)	140	27.08		
**Feelings of loneliness (*N* = 14,226)**					
Seldom/Never	8791 (61.80)	174	1.98	550.30	0.000
Sometimes	4876 (34.27)	312	6.40		
Often	559 (3.93)	117	20.93		

### Prevalence of elderly disabilities

**Fig 1:**
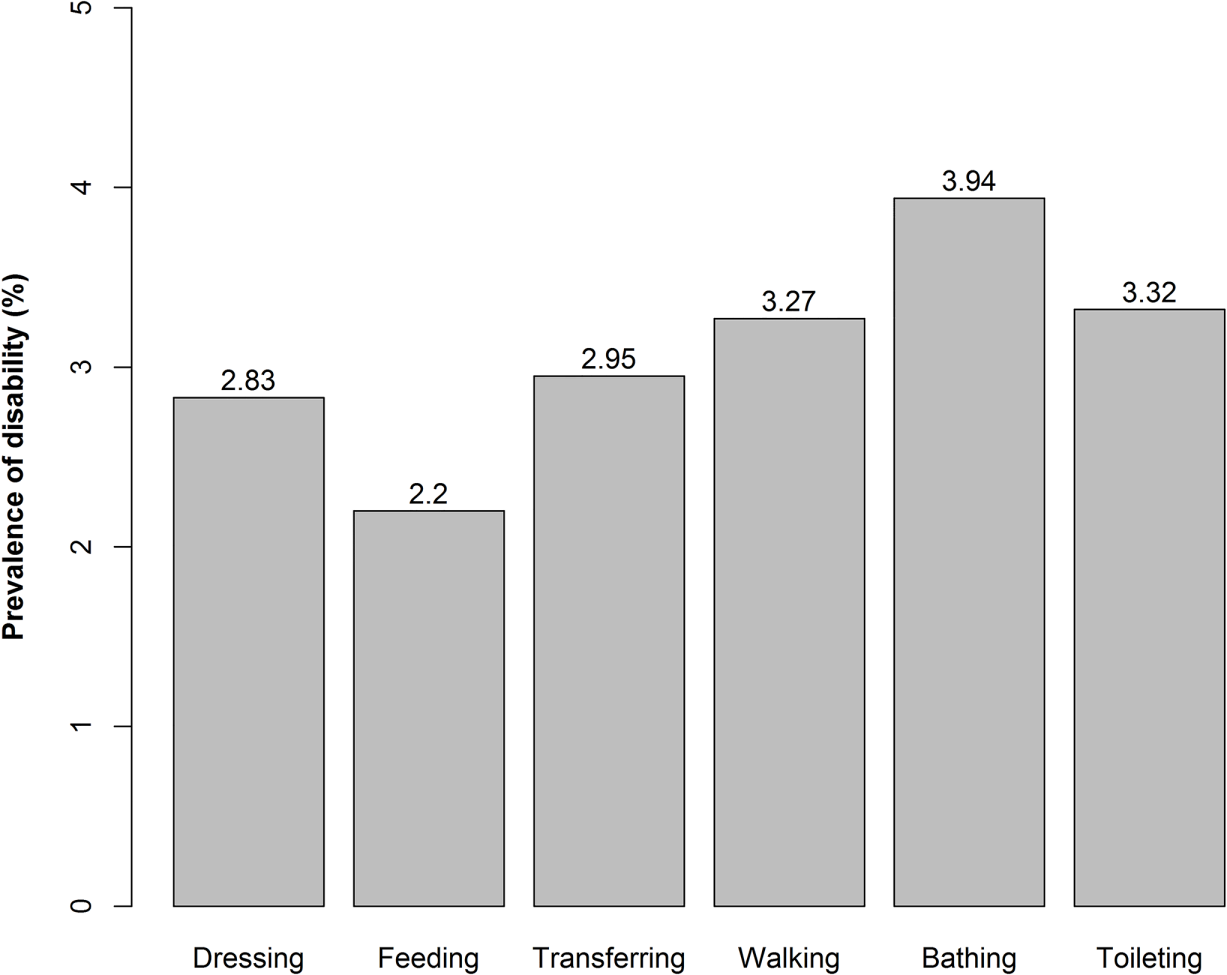
ADL prevalence of disability (n = 14,292). Bars show the disabled prevalence of each ADL item among 14,292 participants. ADL refers to activities of daily living.

As measured by the modified Katz Index Scale, the Cronbach’s alpha coefficient was 0.973. The disability prevalence of 14,292 participants was 4.27%. **Fig 1**showed that the overall prevalence of disability was highest in bathing (3.94%) and the lowest in feeding (2.20%).

The prevalence of elderly disabilities according to different demographic characteristics is shown in **Table 2**. It was obviously higher for those individuals who were ≥80 years of age, female, unmarried, illiterate, in bad economic status, and with bad self-rated health status (i.e. with three or more chronic diseases). Those elderly individuals who reported bad life satisfaction, bad moods, and who often felt lonely had a notably higher prevalence of disability; these percentage prevalences were 19.83%, 27.08%, and 20.93%, respectively. The chi-square test results showed that the differences in disability prevalences were significant in the previously mentioned variables. However, the prevalence of disability was not significant regarding variables related to residence and living arrangements.

### Factors associated with elderly disability

**Table 3**demonstrated the results of binary logistic regression. The elderly who were ≥80 years of age were about five times more likely to have a disability than those 60–69 years of age. The elderly who lived with family had a higher risk of disability than those who lived alone (OR = 3.25). As compared with respondents in good self-rated health, respondents in bad self-rated health were about 74 times as likely to have a disability. Unmarried status, bad economic status, and poor psychological health measures (life satisfaction, mood, and feelings of loneliness) were all risk factors for disability. Residence actually was a protecting factor for elderly who lived in rural (OR = 0.62) settings.

**Table 3:** Binary Logistic Regression analysis of elderly disability (*N* = 14,091).

	OR	95% CI	*P-*value
**Age (years)**			
60–69	Ref.		
70–79	1.94	1.47, 2.56	0.000
≥80	5.80	4.40, 7.66	0.000
**Marital status**			
Married	Ref.		
Unmarried	1.48	1.19, 1.85	0.000
**Living arrangement**			
Alone	Ref.		
With family	3.25	2.34, 4.52	0.000
**Residence**			
Urban	Ref.		
Rural	0.62	0.51, 0.76	0.000
**Personal economic status**			
Income exceeded expenditures	Ref.		
Balance	0.94	0.64, 1.38	0.756
Expenditures exceeded income	2.15	1.49, 3.10	0.000
**Self-rated health**			
Good	Ref.		
Fair	4.00	1.81, 8.82	0.000
Bad	73.95	34.57, 158.22	0.001
**Life Satisfaction**			
Good	Ref.		
Fair	1.13	0.89, 1.45	0.315
Bad	1.84	1.33, 2.54	0.000
**Mood**			
Good	Ref.		
Fair	0.85	0.65, 1.10	0.216
Bad	2.26	1.53, 3.33	0.000
**Feelings of loneliness**			
Seldom/Never	Ref.		
Sometimes	1.58	1.23, 2.04	0.000
Often	1.70	1.15, 2.48	0.008

## Discussion

The population of disabled elderly is rapidly expanding and it is a challenge for individuals, families, and society to undertake the burden of long-term care. Problems related to disabilities in the elderly have attracted both the vigilance and attention of society. This study shows that the prevalence of disability in Xiamen was 4.27% for the population aged ≥60 years. Bathing was the most frequently reported disability and feeding was the least frequently reported disability. In our study, a good reliability was seen relative to the modified Katz Index Scale.

The prevalence of elderly with disabilities in our study was lower as compared with several pertinent Chinese studies in which the same scale of items were used; this includes the latest National Research on Aging Study in 2010 [11] as well as studies conducted by Lin et al [25] and Chou et al [23]. The “Chinese Longitudinal Healthy Longevity Survey (CLHLS)” was a national sample study of elderly ≥65 years of age (primarily ≥80 years of age). We analyzed the latest data from CLHLS (2011) and compared the data with the same five ADL items in our study; we found that the prevalence of elderly with disabilities in our study was lower in every age group (65–69 years of age: 1.39% vs. 1.84%; 70–79 years of age: 3.81% vs. 5.05%; ≥80 years of age: 11.62% vs. 27.59%). This is possibly due to the mild climate and pace of life in Xiamen, which are good for elderly health. The development of health management and elderly care in Xiamen also played positive roles in decreasing the amount of disabilities in elderly individuals. Such differences may also be due to differing definitions of several items. For instance, in CLHLS, the level of “completely dependent” in bathing was defined as an individual (he/she) who can only wash themselves on some parts of their body and need assistance on more than one part of their body. However, in our study, in order to define a more severe disability, the “completely dependent” level was defined as not being able to wash themselves completely and requiring assistance over the whole body.

Many studies in other countries such as those conducted by Ralph et al. (New York, United States of America, 2009) [26], Federman et al. (United States of America, 1996–2001) [27], McLaughlin et al. (United States of America, 2004) [28], Hairi et al. (Malacca, Malaysia, 2007–2008) [29] and Tiago da Silva Alexandre et al. (Sao Paulo, Brazil, 2000) [22] have also used the same items to assess elderly disability. However, they defined the disability in terms of an individual requiring some help or unable to perform one or more ADLs. In our study, the disability was defined as having more severe ADL limitations. One reason for this was that we hoped to compare our results with several studies conducted in China, where there are similar definitions of disability to our study. The other reason was that we would like to identify elderly individuals who were totally dependent on other individuals help and required long-term care solutions; a large number of Chinese elderly have ADL limitations and relatively limited health care resources. If we applied their definition of disability in our study, the prevalences of disability in people ≥ 60 years of age and ≥ 65 years of age were 12.55% (1793/14,292) and 15.55% (1648/10,598) respectively, still lower than these other reports.

Many demographic factors were associated with disability. Elderly people who lived alone [30,31] or in rural [11,13–15] residences had a higher risk of disability due to a lack of support from family or social services. Interestingly, we found that the elderly who lived with family members or lived in urban areas had a greater risk of disability in our study. As for the variable living arrangements, we found that the prevalence of disability was higher in elderly people who either lived with children (8.27%) or lived with others (9.68%) than for those who lived alone (3.86%). This information was consistent with several previous research studies [16,17] which showed that elderly individuals who had no spouse and either lived with children or other individuals had a worse outcome than the elderly who lived alone. The interpretation was that elderly individuals who had a good health status and were financially independent preferred to either live alone or with their spouse [17]. In other words, elderly individuals either lived with children or other individuals possibly because they required more support and care from their families. The authors also found that the proportion of elderly individuals who lived alone in rural (14.6%) residences was almost double as compared with the elderly who lived in urban (7.4%) residences; hence, living arrangements may be a confounder to residence. This may also explain why differences were not significant between the prevalence of disability and different residential living arrangements in chi-square tests; however, binary logistic regression results showed that the elderly who lived in urban settings or lived with family had a higher risk of disability. Additionally, female gender, older age, unmarried status, lower educational status, and bad economic status were all risk factors for disability, similar to many other research studies [15,16,26].

The variables related to health status all strongly influence disability. In our study, self-rated health was most strongly associated with disability. The elderly who had worse self-rated health measurements had a higher prevalence of disability, similar to some previous studies [16,27]. Self-rated health is a subjective assessment of individual health status; it is a reliable predictor of disability and future mortality [32,33]. The number of chronic disease is one major risk related to disability as proven in many countries [34]. Parallel to many studies, we found the elderly suffered more kinds of chronic disease and appeared to have worse functional status [13,16,26, 35]. We must emphasize that several chronic diseases are strongly associated with poor physical function, such as cardiovascular disease, dementia, arthritis, and other musculoskeletal diseases [34,36]. The top three chronic diseases in our study were hypertension (30.10%), arthritis (24.76%), and lumbar cervical disease (10.60%), all of which are major risks for disability, similar to other research studies [37,38]. Although this variable number of chronic diseases is important, it did not enter the model of binary logistic regression. This may be due to existing multicollinearity among different variables. For instance, the effect related to the number of chronic diseases may be covered by the self-rated health and age in the binary logistic regression model, because the elderly or in those in bad self-rated health may suffer more kinds of chronic diseases. Poor psychosocial condition was also shown to be a risk for disability in the disablement model developed by Verbrugge et al. [39]; however, to the best of our knowledge, it is rarely an influential factor requiring further study. The elderly in our study who had poor life satisfaction, bad mood, and often felt lonely showed a higher prevalence of disability. The World Health Organization emphasized that people who had poor mental and psychosocial conditions were more likely to suffer disability, because they had barriers related to accepting education, finding employment, and therefore, full participation in their societies [40].

The prevalence of disability in our municipal-level study was lower than national studies [11]; according to this prevalence model, in 2013, there were about 11,300 disabled elderly in Xiamen city. Thus, we need to develop several appropriate policies to care for the large numbers of disabled elderly. Long-term care is important for elderly with disabilities; it has been placed at the forefront of the national political agenda in many countries such as Germany, Japan, and the United States [41]. However, in China, municipal level assessment remains in the initial stage and long-term care insurance has not been established. Most of the elderly Chinese with disabilities are cared for by their family caregivers and consequently impose a large burden for their families. The government should consider giving some assistance, such as establishing a hospital for geriatric patients and developing a specialized guide for rehabilitation of disabled individuals and their families in either community-based or home settings. As many factors are associated with disabilities, programs should be effectively developed in order to prevent disabilities in older adults. These programs should focus on strengthening management of chronic diseases and intensifying mental health education.

There are several limitations associated with this study. First, the cross-sectional data limit interpretation of the results, making it difficult to draw causal conclusions. For instance, the relationship noted between disability and living arrangements could be reversed. Second, our study was conducted only in Xiamen city, so nationwide generalizations might be limited. Third, although we tried to retrieve a representative sample, randomization was incomplete at the community level. Finally, functional status of the elderly was assessed by self-reported measurements; we did not survey caregivers or care providers to obtain a more comprehensive picture of elderly disabilities. Future assessments will need to conduct longitudinal studies to assess policy trends and key stakeholders. Thus, urgent action is necessary to care for these vulnerable and disabled older adult populations.

## References

[1] Chen S (2009) Aging with Chinese characteristics: a public policy perspective. Ageing Int 34: 172–188.

[2] Zhao P, Dai M, Chen W, Li N (2010) Cancer trends in China. Jpn J Clin Oncol 40: 281–285. 10.1093/jjco/hyp187 20085904

[3] Woo J, Kwok T, Sze FK, Yuan HJ (2002) Ageing in China: health and social consequences and responses. Int J Epidemiol 31: 772–775. 12177017 10.1093/ije/31.4.772

[4] Chen F, Liu G (2009) Population aging in China International handbook of population aging. Springer Netherlands: 157–172.

[5] Zhong H (2011) The impact of population aging on income inequality in developing countries: Evidence from rural China. Chin Econ Rev 22: 98–107.

[6] Grigsby JS, Olshansky SJ (1989) The demographic components of population aging in China. J Cross-cult Gerontol 4: 307–334. 12283126

[7] National Bureau of Statistics of People’s Republic of China (2014) National Economic and Social Development Statistics Bulletin, People's Republic of China in 2013. Beijing.

[8] Peng X, Song S, Sullivan S, Qiu J, Wang W (2010) Ageing, the urban-rural gap and disability trends: 19 years of experience in China-1987 to 2006. PLoS One 5: e12129 10.1371/journal.pone.0012129 20730089 PMC2921329

[9] Feng W, Mason A (2007) Population aging in China: Challenges, opportunities, and institutions. Population in China at the Beginning of the 21st Century: 177–196.

[10] Wang Y, Wang J, Maitland E, Zhao Y, Nicholas S, Lu M (2012) Growing old before growing rich: inequality in health service utilization among the mid-aged and elderly in Gansu and Zhejiang Provinces, China. BMC Health Serv Res 12: 302 22947369 10.1186/1472-6963-12-302 PMC3447639

[11] The Research Group of China Research Center on Aging (2011) Research on situation of urban and rural disabled elderly. Disability research 1: 11–16.

[12] Yang M, Ding X, Dong B (2014) The measurement of disability in the elderly: a systematic review of self-reported questionnaires. J Am Med Dir Assoc 15: 150:e1-e9. 10.1016/j.jamda.2013.10.004 24314698

[13] Gong C, Bin C, Lei Z (2010) Study on the prevention and strategy of disability in china. Procedia-social and behavioral sciences 2: 6906–6913.

[14] Kaneda T, Zimmer Z, Zhang K (2010) Explaining the urban-rural divergence in disability among older Chinese using a large scale national dataset Institute of Public and International Affairs (IPIA) Working Paper Series. Salt Lake City, Utah: IPIA, University of Utah. April 19: 4–19.

[15] Liu J (2008) Disability among the elderly in China: Levels, trends, composition and determinants. International Institute for Applied Systems Analysis. Resource document. Available: http://www.iiasa.ac.at/Research/HGC/docs/IIASA% 20Final% 20Report.20.

[16] Wang H, Chen K, Pan Y, Jing F, Liu H (2013) Associations and impact factors between living arrangements and functional disability among older Chinese adults. PLoS One 8: e53879 10.1371/journal.pone.0053879 23342030 PMC3544765

[17] Wang D, Zheng J, Kurosawa M, Inaba Y, Kato N (2009) Changes in activities of daily living (ADL) among elderly Chinese by marital status, living arrangement, and availability of healthcare over a 3-year period. Environ Health Prev Med 14: 128–141. 10.1007/s12199-008-0072-7 19568857 PMC2684772

[18] Xiamen Municipal Bureau of Statistics (2014) National Economic and Social Development Statistics Bulletin of Xiamen city in 2013 Beijing: China Statistics Press.

[19] Fang J (2007) Statistical methods for biomedical research Beijing: Higher Education Press: 267–268.

[20] World Health Organization (2001) The International Classification of Functioning, Disability and Health. Geneva, Switzerland.

[21] Rodgers W, Miller B (1997) A comparative analysis of ADL questions in surveys of older people. J Gerontol B Psychol Sci Soc Sci 52(Special Issue): 21–36. 9215355 10.1093/geronb/52b.special_issue.21

[22] Alexandre TS, Corona LP, Nunes DP, Santos JL, Duarte YA, Lebrão ML (2012) Gender differences in incidence and determinants of disability in activities of daily living among elderly individuals: SABE study. Arch Gerontol Geriatr 55: 431–437. 10.1016/j.archger.2012.04.001 22546518

[23] Chou KL, Leung JC (2008) Disability trends in Hong Kong community-dwelling Chinese older adults 1996, 2000, and 2004. J Aging Health 20: 385–404. 10.1177/0898264308315852 18378722

[24] Katz S, Ford AB, Moskowitz RW, Jackson BA, Jaffe MW (1963) Studies of illness in the aged: the index of ADL: a standardized measure of biological and psychosocial function. JAMA 185: 914–919. 14044222 10.1001/jama.1963.03060120024016

[25] Lin H, Zhang TH, Tang H, Wang CB, Liu N, Zhang XP, et al (2002) Analysis of influential factors of activities of daily life of the elderly. Chin Health Serv Manag 18: 495–497.

[26] Ralph NL, Mielenz TJ, Parton H, Flatley AM, Thorpe LE (2013) Peer Reviewed: Multiple chronic conditions and limitations in activities of daily living in a community-based sample of older adults in New York City, 2009. Preventing chronic disease 10. 10.5888/pcd10.130159 PMC3843532 24286273

[27] Federman AD, Penrod JD, Livote E, Hebert P, Keyhani S, Doucette J, et al (2010) Development of and recovery from difficulty with activities of daily living: an analysis of national data. J Aging Health 22: 1081–1098. 10.1177/0898264310375986 20660637

[28] McLaughlin SJ, Connell CM, Heeringa SG, Li LW, Roberts JS (2010) Successful aging in the United States: prevalence estimates from a national sample of older adults. J Gerontol B Psychol Sci Soc Sci 65: 216–226. 10.1093/geronb/gbp101 PMC2981444 20008481

[29] Hairi NN, Bulgiba A, Cumming RG, Naganathan V, Mudla I (2010) Prevalence and correlates of physical disability and functional limitation among community dwelling older people in rural Malaysia, a middle income country. BMC Public Health 10: 492 10.1186/1471-2458-10-492 20716377 PMC2933720

[30] Gao L (2011) The health condition and its determinants of the elderly population in Shandong Province Shandong Univercity: Jinan, China Available: http://cdmd.cnki.com.cn/Article/CDMD-10422-1011170391.htm.

[31] Kharicha K, Iliffe S, Harari D, Swift C, Gillmann G, Stuck AE (2007) Health risk appraisal in older people 1: are older people living alone an ‘at-risk’group? Br J Gen Pract 57: 271–276. 17394729 PMC2043328

[32] Idler EL, Benyamini Y (1997) Self-rated health and mortality: a review of twenty-seven community studies. J Health Soc Behav 21–37. 9097506

[33] Sun W, Watanabe M, Tanimoto Y, Shibutani T, Kono R, Saito M, et al (2007) Factors associated with good self-rated health of non-disabled elderly living alone in Japan: a cross-sectional study. BMC Public Health 7: 297 17949511 10.1186/1471-2458-7-297 PMC2186322

[34] Lafortune G, Balestat G (2007) Trends in severe disability among elderly people: assessing the evidence in 12 OECD countries and the future implications. OECD Health Working Papers, No. 26, OECD Publishing. Available: 10.1787/217072070078

[35] Arias-Merino ED, Mendoza-Ruvalcaba NM, Ortiz GG, Velázquez-Brizuela IE, Meda-Lara RM, Cueva-Contreras J (2012) Physical function and associated factors in community-dwelling elderly people in Jalisco, Mexico. Arch Gerontol Geriatr 54: e271–e278. 10.1016/j.archger.2012.02.010 22440759

[36] Hairi NN, Bulgiba A, Mudla I, Hiong TG (2012) Physical function in older people In: Geriatrics. Atwood C (ed.); InTech Available: http://www.intechopen.com/books/geriatrics/physical-function-in-older-people

[37] Klijs B, Nusselder WJ, Looman CW, Mackenbach JP (2011) Contribution of chronic disease to the burden of disability. PLoS One 6: e25325 10.1371/journal.pone.0025325 21966497 PMC3178640

[38] Caskie GI, Sutton MC, Margrett JA (2010) The relation of hypertension to changes in ADL/IADL limitations of Mexican American older adults. J Gerontol B Psychol Sci Soc Sci 65: 296–305. 10.1093/geronb/gbq001 PMC3107028 20110317

[39] Verbrugge LM, Jette AM (1994) The disablement process. Soc Sci Med 38: 1–14. 8146699 10.1016/0277-9536(94)90294-1

[40] Chan M (2010) Mental health and development: targeting people with mental health conditions as a vulnerable group. World Health Organization. Available: http://www.who.int/mental_health/policy/development/mh_devel_targeting_summary.pdf, 2010.

[41] Reichert M, Naegele G, Katz R, Lowenstein A, Halperin D (2014) Long-term care needs and long-term care policy: comparing Germany and Israel. Family and Health: Evolving Needs, Responsibilities, and Experiences (Contemporary Perspectives in Family Research, Volume 8B). Emerald Group Publishing Limited 8; 131–167.

